# Natural Compound Histone Deacetylase Inhibitors (HDACi): Synergy with Inflammatory Signaling Pathway Modulators and Clinical Applications in Cancer

**DOI:** 10.3390/molecules21111608

**Published:** 2016-11-23

**Authors:** Hélène Losson, Michael Schnekenburger, Mario Dicato, Marc Diederich

**Affiliations:** 1 Laboratoire de Biologie Moléculaire et Cellulaire du Cancer (LBMCC), Hôpital Kirchberg, 9 Rue Edward Steichen, Luxembourg L-2540, Luxembourg; helene.losson@lbmcc.lu (H.L.); michael.schnekenburger@lbmcc.lu (M.S.); mdicato@gmail.com (M.D.); 2 Department of Pharmacy, College of Pharmacy, Seoul National University, Building 29 Room 223, 1 Gwanak-ro, Gwanak-gu, Seoul 08826, Korea

**Keywords:** histone deacetylase inhibitors, anticancer drugs, vorinostat, romidepsin, belinostat, panobinostat, histone acetylation, nuclear factor-κB modulation

## Abstract

The remarkable complexity of cancer involving multiple mechanisms of action and specific organs led researchers Hanahan and Weinberg to distinguish biological capabilities acquired by cancer cells during the multistep development of human tumors to simplify its understanding. These characteristic hallmarks include the abilities to sustain proliferative signaling, evade growth suppressors, resist cell death, enable replicative immortality, induce angiogenesis, activate invasion and metastasis, avoid immune destruction, and deregulate cellular energetics. Furthermore, two important characteristics of tumor cells that facilitate the acquisition of emerging hallmarks are tumor-promoting inflammation and genome instability. To treat a multifactorial disease such as cancer, a combination treatment strategy seems to be the best approach. Here we focus on natural histone deacetylase inhibitors (HDACi), their clinical uses as well as synergies with modulators of the pro-inflammatory transcription factor signaling pathways.

## 1. Introduction

Epigenetic modifications can be the cause of each of the cancer hallmarks. Indeed, the term “epigenetic” refers to the change of a gene’s expression without a change in its DNA sequence. Therefore, epigenetic modifications can regulate the expression of each gene implicated in cell tumorigenesis. Epigenetic modifications of DNA methylation, histone modification marks, and small RNA-mediated gene silencing are dynamic and potentially reversible changes [1]. We will focus on histone deacetylases (HDACs) that are implicated in histone modification. Increasing interest has been centered on the development of histone deacetylase inhibitors (HDACi) for cancer treatment, as they can modulate gene expression and the activity of numerous non-histone proteins. However, HDACi toxicity and limited clinical benefit in patients with solid tumors, have led to the investigation of combination treatments with other cancer therapeutics [2].

Epidemiological studies showed that regular consumption of vegetables and fruits is associated with a reduced risk of chronic diseases such as cancer. Natural products have been inevitably used as therapeutic agents. Moreover, most medicinal substances available today have their origin in natural products [2].

## 2. Histone Deacetylases and Their Classification

**Figure 1:**
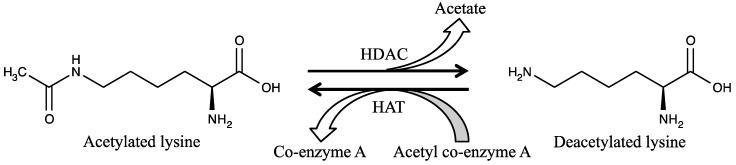
Acetylation and deacetylation reactions of lysine. The acetylation reaction is catalyzed by histone acetyltransferases (HATs) using acetyl co-enzyme A as an acetyl group donor. Deacetylation, on the other hand, is catalyzed by histone deacetylases (HDACs).

Among epigenetic modifications, histone acetylation has been the best studied. Histone acetylation status is controlled by histone acetyltransferases (HATs) and HDACs, which add or remove, respectively, an acetyl group on ε-amino groups of lysine residues in histone N-terminal regions [3]. Figure 1 shows acetylation and deacetylation reactions.

Histone hypoacetylation is associated with gene silencing because of an electrostatic interaction between the positive charge of hypoacetylated histones and negative charge of DNA phosphate, which maintains chromatin as a condensed structure. Moreover, the activity of non-histone proteins, such as signal transduction mediators, chaperone proteins, transcription factors, structural proteins, and inflammation mediators, is also dependent on their acetylation status regulated by HATs and HDACs. Consequently, a change in acetylation status has consequences for protein-DNA interactions, protein-protein interactions, and protein stability [4].

**Figure 2:**
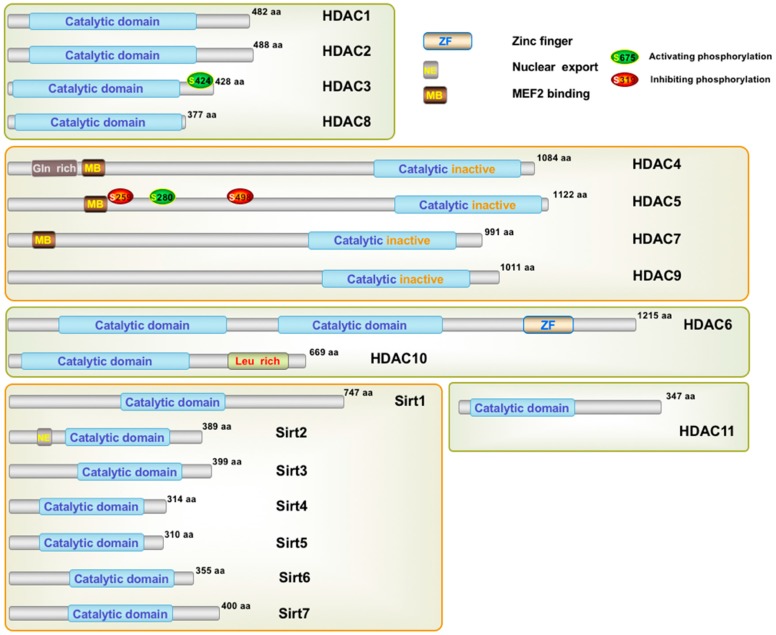
The molecular structure of human histone deacetylases (scheme realized with ScienceSlides 2016; VisiScience Inc., Chapel Hill, NC, USA).

In humans, 18 HDACs, divided into two families, have been identified so far based on their homology to yeast deacetylases. One family requires zinc ion (Zn^2+^) as a cofactor for deacetylase activity and includes HDAC1 to 11. A sequence homology was found between the yeast deacetylase reduced potassium dependency-3 (Rpd3) and HDAC1, 2, 3 and 8; and similarly, between the yeast histone deacetylases-1 (Hda1) and HDAC4, 5, 6, 9 and 10. HDAC11 shares sequence identity with both yeast deacetylases. The second HDAC family requires the cofactor nicotinamide adenine dinucleotide (NAD^+^) and includes seven members, the sirtuins (SIRTs) 1 to 7 that are related to the yeast deacetylase silent information regulator-2 (Sir2) [2]. The two HDAC families are divided into four classes based on their size and cellular location. Class I (HDAC1, 2, 3 and 8) and class IV (HDAC11) are mainly found in the nucleus, while class IIb (HDAC6 and 10) is primarily found in the cytoplasm. Class IIa (HDAC4, 5, 7 and 9) is found shuttling between the nucleus and cytoplasm. Class III enzymes (sirtuins 1–7) are localized in the nucleus, cytoplasm, and mitochondria [5]. Table 1 summarizes the classification, protein size, implicated co-factor, cellular localization, some physiological functions, and expression of these HDACs [6,7]. Figure 2 represents the molecular structure of the different classes of HDACs.

## 3. Histone Deacetylases and Cancer

According to recent studies, altered acetylation levels and HDAC enzyme dysfunctions are linked to numerous human diseases including cancer. It has been shown that expression of HDACs is increased in hematological cancers and solid tumors, and is correlated with a poor prognosis. HDACs are involved in deacetylation of not only chromatin proteins, which can alter the regulation of gene transcription, but also non-histone proteins that control apoptosis and/or cell cycle progression and differentiation [8]. The involvement of HDACs at these regulation levels explains why they can be strongly implicated in the acquisition of malignant phenotypes. We will next focus on the role of each HDAC class in cancer.

### 3.1. Class I

HDACs that belong to class I induce cell proliferation but inhibit differentiation and apoptosis [9]. Some researchers have studied the expression of HDAC genes in different cancers. In this manuscript, *HDAC* in italics designate the corresponding gene. A *HDAC1* overexpression has been found in Hodgkin’s lymphoma, ovarian, prostate, and gastric cancer [4]. Furthermore, an increase in HDAC1 expression has been reported in gastrointestinal and prostate cancer, breast carcinomas [9], colon adenocarcinoma [7], and chronic lymphocytic leukemia (CLL) [10]. However, a downregulation of HDAC1 has been observed in colorectal cancer [4]. Overexpression of HDAC2 has been observed in uterine, cervical, and gastric cancers [9], while *HDAC2* overexpression was detected in ovarian cancer and Hodgkin’s lymphoma [4]. In some cancers, such as colon, endometrial, and gastric cancers, a truncating *HDAC2* mutation has been found [4]. *HDAC3* overexpression has been observed in Hodgkin’s lymphoma [4], colon cancer [9], and CLL [10]. Moreover, *HDAC3* overexpression has been found in ovarian and lung cancers [4]. However, an increased expression of HDAC1 and 3 was paradoxically related to disease-free survival in invasive breast cancer patients [7].

### 3.2. Class II

Class II HDACs induce tumor angiogenesis [9]. However, reduced expression of class II HDACs is related to a poor clinical outcome in non-small-cell lung cancer patients [7]. *HDAC4* missense mutations have been observed in breast and colorectal cancers, while this HDAC is overexpressed in breast cancer [4]. Nevertheless, *HDAC4* has been shown to be downregulated in lung and colon cancers [4].

Interestingly in colorectal cancer, we find an *HDAC5* downregulation but an *HDAC7* overexpression, which is also observed in pancreatic cancer [4]. An overexpression of HDAC7 and HDAC9 has been detected in CLL [10]. HDAC6 specifically increases cell motility that results in distant metastasis [9]. HDAC6 has been reported to be overexpressed in acute lymphoblastic leukemia (ALL), acute myeloid leukemia (AML), breast cancer, CLL, cutaneous T-cell lymphoma (CTCL), hepatocellular carcinoma, oral squamous cell carcinoma, and ovarian and urothelial cancers. Paradoxically its overexpression is correlated with longer survival in CLL and CTCL [11]. Finally, HDAC10 overexpression has been reported in CLL [10].

### 3.3. Class III

An overexpression of sirtuin (SIRT)1 has been reported in CLL [10], AML, skin, colon, and prostate cancers [12]. Nevertheless, a *SIRT1* downregulation has been found in colorectal cancer [13]. Furthermore, a decrease in SIRT6 expression has been observed in liver cancer [14], while its overexpression has been found in CLL [10]. SIRT7 is overexpressed in breast cancer [15].

### 3.4. Class IV

HDAC11 protein does not seem to be implicated in tumorigenesis [4]. Table 2 summarizes the variation observed in HDAC protein and gene expression levels and their implication in specific cancers.

## 4. Natural Compound Histone Deacetylase Inhibitors

Altogether natural compounds provide pleiotropic and potent inhibitors of all hallmarks of cancer [17,18,19,20]. Overall, it becomes mandatory to undertake a careful selection of natural compounds regarding specificity, drug-like characteristics and pharmacokinetic properties including pharmacologically relevant active concentration as well as potential side effects.

Natural compounds and their hemisynthetic derivatives of terrestrial [17] and marine origins [20] are considered potent anticancer as well as chemopreventive agents [21]. These compounds were shown to target multiple cancer cell signaling pathways leading to induction of various forms of cell death including apoptotic, autophagic [22] and more recently so-called non-canonical types of cell death [23].

Moreover, natural compounds provide pharmacological scaffolds that modify the epigenetic regulation of gene expression [19], allow cell-type specific differentiation with the aim to reprogram differentiation pathways [18] and act as inhibitors of inflammation [24]. Many natural compounds seem to interfere with a majority of molecular mechanisms involving proliferation and cell death (polyphenolic compounds, for example fisetin [25], curcumin [26], resveratrol [27], chalcones [28] or flavonoids [29]).

### 4.1. Natural Compound Scaffolds of Clinically Used HDAC Inhibitors

HDACi belong to a large family divided into five main classes according to their structure including hydroxamic acids, benzamides, cyclic peptides, short-chain fatty acids, and depsipeptides [3]. Interestingly, the four clinically approved HDACi are based on scaffolds originally discovered in microorganisms.

**Figure 3:**
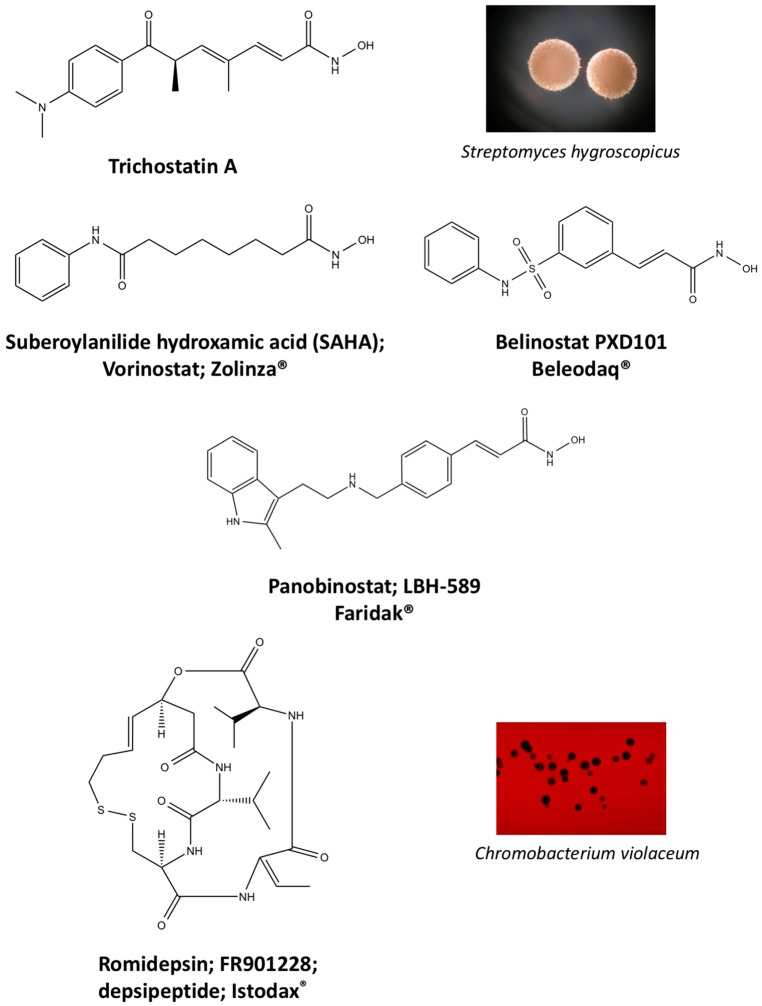
Molecular structures of clinically approved histone deacetylase inhibitors. (Trichostatin A is not clinically approved but added here as a precursor of a structural precursor of clinically used hydroxamate-derivatives).

In 1976, Tsuji et al. [30] described a natural compound, (*R*)-trichostatin A (TSA) (Figure 3), a naturally occurring hydroxamate antibiotic, originally extracted from *Streptomyces hygroscopicus*. TSA can trigger cell differentiation and interrupt the cell cycle of both normal and cancer cells together with the accumulation of acetylated histones [31] and can be considered as a structural precursor of clinically used hydroxamate-derivatives. Related synthetic hydroxamate suberoylanilide hydroxamic acid (SAHA; Vorinostat) (Figure 3), originally derived from hexamethylene bisacetamide (HMBA), were developed later [32] and eventually reached clinical applications. Similarly, synthetic compound PXD101 (Belinostat) (Figure 3) also belongs to the class of hydroxamates but inhibits HDACs in a low nanomolar range [33]. Furthermore, Novartis developed Panobinostat (Figure 3), a cinnamic hydroxamate [34].

In 1994, Ueda et al. [35] purified FR90122 (Figure 3), an antitumor bicyclic depsipeptide produced by *Chromobacterium violaceum* No. 968. Its HDAC inhibitory capacity was described by Nakajima et al. [36] leading to cell cycle arrest in G1 and G2/M stages.

### 4.2. Experimentally Used Natural Compound Scaffolds without Clinical Utilization

**Figure 4:**
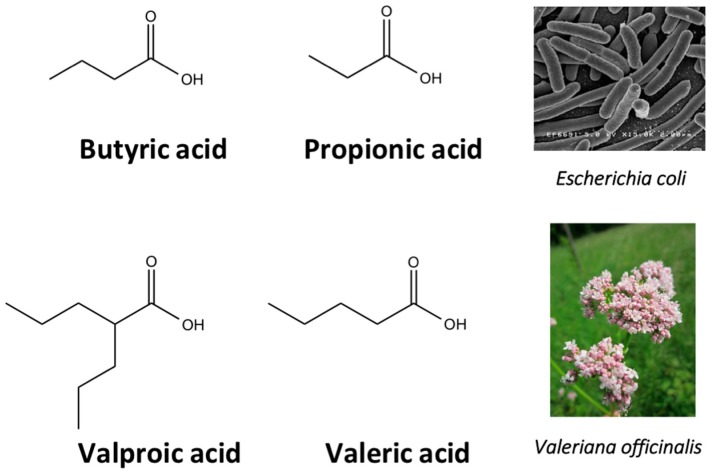
Molecular structures of short chain fatty acid HDACi scaffolds of natural origins.

In 1977, Riggs et al. [37] described for the first time the effect of butyrate on histone modifications in HeLa and Friend erythroleukemia cell lines. In 1980, McKnight et al. [38] also investigated propionate, which had a lesser effect, on histone deacetylation in chick oviduct. Both natural compounds are active at millimolar levels and were initially discovered to be synthesized by colonic bacteria and contribute to the survival of colon cells otherwise undergoing autophagy. McKnight et al. also speculated that other proteins besides histones could be the target of acetylation changes and thus contribute to regulation of gene expression. Beyond cancer treatment, the effect of butyrate on colon cancer cells could also contribute to its cancer preventive potential [39]. Longer chained synthetic HDACi from the family of the fatty acids, valproic acid (VPA), is a clinically approved drug against epilepsy since the 1960s [40]. More recently, potent HDAC inhibition by VPA by binding to the active site of HDAC triggered differentiation of human leukemia cells [41]. This compound also has structural resemblance with the natural compound valeric acid extracted from *Valeriana officinalis* (Figure 4).

In addition to the already mentioned natural compounds and their derivatives, numerous other natural compounds from both terrestrial and marine origin were described in the literature without apparent clinical applications yet (Table 3).

## 5. Clinically Approved Histone Deacetylase Inhibitors

Overexpression of various HDAC proteins is frequently reported in numerous cancers. In such cases, inhibitors of HDACs that reverse the malignant phenotype have emerged as promising anticancer therapeutics. The many biological effects due to HDAC inhibition are still unknown, as some important histones and non-histone proteins are regulated by acetylation. However, HDACi mainly induce hyperacetylation and consequently gene expression; they are implicated in chromatin stability, mitosis, and DNA repair mechanisms [4]. Even though a similar accumulation of acetylated histones has been shown after treatment of normal and tumor cells with HDACi, healthy cells appear to be much less sensitive to their apoptotic and growth inhibition effects than tumor cells [4]. All these characteristics seem to confirm the strong potential of HDACi in cancer treatment. To date, four HDACi have been approved by the United States Food and Drug Administration (USFDA) for cancer therapy: vorinostat, romidepsin, belinostat, and panobinostat. Three pharmacophores are found in class I and II HDACi, despite structural diversity: (1) A cap group which is in contact with residues on the rim of the binding pocket able to obstruct the entrance of the substrate; (2) a Zn^2+^ binding domain (ZBD); (3) a tail or linker that is a saturated or unsaturated aliphatic chain that can mimic the side chain of lysine [3,8].

### 5.1. Vorinostat

The first drug approved by USFDA in October 2006 for CTCL treatment was vorinostat (Figure 3) also known as SAHA or Zolinza^®^. Vorinostat is a linear hydroxamate compound, developed by Merck & Co. Inc., that inhibits class I, II, and IV HDACs [9]. An objective response rate of 30% was determined after a phase II trial in which 74 patients were given a daily oral administration of vorinostat (400 mg) [2]. Furthermore, a significant 31% response was obtained with continuous daily administration compared to an intermittent dosing (9%) [2]. However, severe side effects such as anemia and thrombocytopenia were reported, more frequently in patients who received vorinostat by intravenous administration [9]. Vorinostat appears to be useful in the treatment of some others diseases besides CTCL. Indeed, in a phase I trial, a clinical benefit was observed in seven of 41 patients with myelodysplastic syndromes (MDS) and advanced leukemia treated with vorinostat. According to clinicaltrials.gov, vorinostat is also being studied in 39 ongoing clinical trials as a single therapeutic agent for various cancers such as leukemia, non-small-cell lung cancer, multiple myelomas, MDS, and breast, pelvic and prostate cancers [70].

### 5.2. Romidepsin

Romidepsin (Figure 3), also known as FK228, FR901228, depsipeptide, or Istodax^®^, was the second drug approved by USFDA in November 2009 for CTCL, and in November 2011 for the treatment of peripheral T-cell lymphoma (PTCL). Romidepsin is a cyclic tetrapeptide isolated from the fermentation product of *Chromobacterium violaceum*, developed by Gloucester Pharmaceuticals, and it inhibits predominantly class I HDACs [9,71]. An overall response rate of 34% was determined after two phase II trials in which romidepsin was administered by intravenous infusion to 96 CTCL patients [2]. An objective response rate of 25% was obtained in PTCL patients [9]. However, side effects were reported such as nausea, vomiting, myelotoxicity, asthenia, and cardiac toxicity [72]. Currently, 34 ongoing clinical trials are studying the effect of romidepsin as a single agent on many other cancers such as leukemia, melanoma, and bladder, colorectal, breast, ovarian and prostate cancers (clinicaltrials.gov) [70].

### 5.3. Belinostat

The third drug (Figure 3) approved by USFDA in July 2014 for relapsed or refractory PTCL was belinostat (also named PXD101 or Beleodaq^®^). It is a hydroxamic acid-based compound developed by Spectrum Pharmaceuticals, that inhibits class I and II HDACs [73]. An overall response rate of 26% was determined from clinical responses obtained after a multicenter, single arm trial on 120 PTCL patients who were refractory or had relapsed after their first treatment [6]. However, adverse events were reported, mainly fatigue, nausea, vomiting, diarrhea, constipation, phlebitis, headache, and dyspnea [72]. Belinostat is used in 15 ongoing clinical trials as a single agent for the treatment of cancers such as CTCL, MDS, multiple myelomas, Burkitt lymphoma, and solid tumors as in fallopian tube cancer [70].

### 5.4. Panobinostat

Panobinostat (LBH-589 or Faridak^®^) (Figure 3) was the fourth drug approved by USFDA in February 2015 for the treatment of multiple myelomas [9]. It is also a hydroxamic acid-based compound developed by Novartis, but that inhibits class I, II and IV HDACs [74]. Panobinostat has shown an objective response of 27% [9]. However, it induces severe diarrhea and cardiac toxicities [75]. Panobinostat is being studied in 50 clinical trials as a single agent for the treatment of other cancers such as CTCL, AML, Hodgkin’s lymphoma, MDS, thyroid carcinoma, and colorectal and prostate cancers [70].

The four approved HDACi are indicated for hematological cancers, such as CTCL, PTCL, and multiple myelomas, but not for solid tumors. Early results of clinical trials including solid tumor patients have shown that HDACi are more efficient in the treatment of hematological cancers than solid tumors. Currently, the exact reasons for this are unknown, but some observations suggest that it may be explained by the instability of HDACi and hence their inability to reach solid tumor sites [6].

## 6. Combined Treatments of Histone Deacetylase Modulators and NF-κB Inhibitors

### 6.1. Post-Translational Modifications of NF-κB

**Figure 5:**
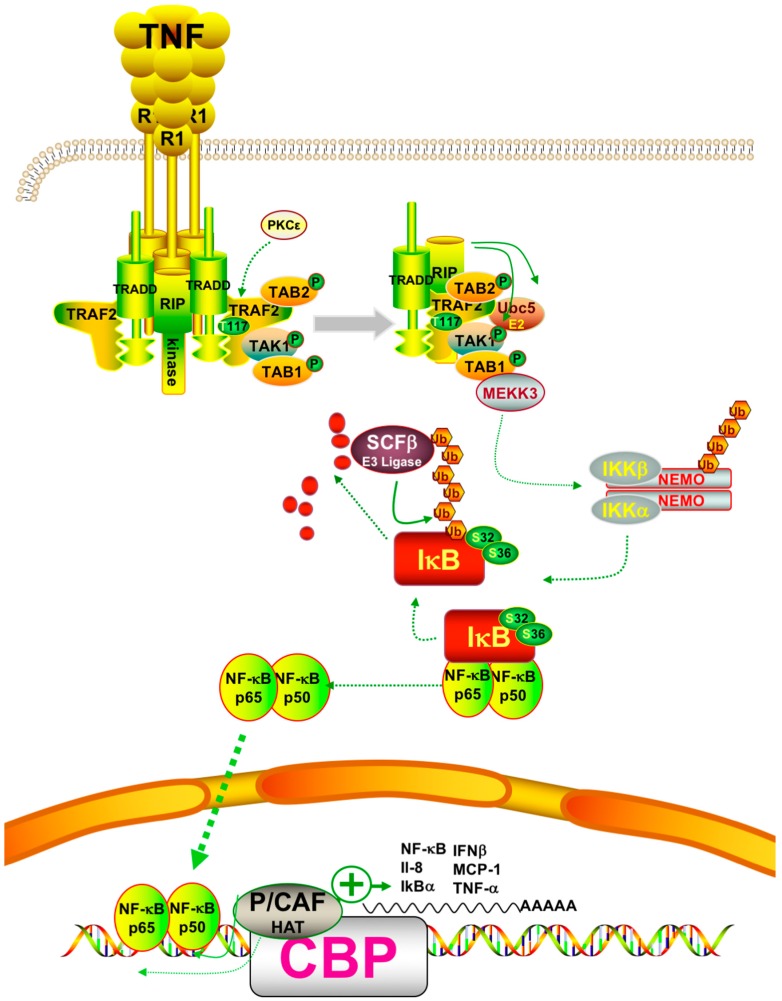
Upstream signal transduction pathway leading to activation of canonical p50/p65 NF-κB upon stimulation by tumor necrosis factor (TNF)-α (see text for details, scheme realized with ScienceSlides). Tumor necrosis factor receptor type 1-associated death domain (TRADD); TNF receptor-associated factor (TRAF); transforming growth factor β-activated kinase (TAK); TGF-β-activated kinase 1 and MAP3K7-binding protein (TAB); Ubiquitin-conjugating (enzyme) (Ubc); IκB kinase (IKK); Skp, Cullin, F-box (containing complex) (SCF); inhibitor of κB (I-κB); nuclear factor (NF)-κB, P300/CBP-associated factor (P/CAF); interleukin (Il); interferon (INF); monocyte chemotactic protein-1 (MCP); ubiquitin (Ub); cAMP-response element-binding protein binding protein (CBP); histone acetyltransferase (HAT); receptor-interacting protein (RIP); protein kinase (PK); NF-κB essential modulator (NEMO); mitogen-activated protein kinase kinase kinase 3 (MEKK3).

Whereas the basic nuclear factor (NF)-κB cell signaling pathways are well known and investigated [76], synergistic interaction with epigenetic modifications and especially histone-mediated modifications remain a topic of investigation. Briefly, canonical activation of NF-κB takes place after stimulation of the tumor necrosis factor (TNF)-α receptor, triggering the formation of a signalosome comprising tumor necrosis factor receptor type 1-associated death domain (TRADD) adaptor protein, receptor-interacting protein (RIP) kinase, TNF receptor-associated factor (TRAF)2, transforming growth factor (TGF)-β-activated kinase 1 and MAP3K7-binding protein (TAB) 1 and 2 as well as TGF-β-activated kinase (TAK)1 leading to phosphorylation and activation of mitogen-activated protein kinase kinase kinase (MEKK)3. This kinase phosphorylates and activates IκB kinase (IKK) which in turn phosphorylates IκB, the natural inhibitor of NF-κB [77]. Upon phosphorylation, IκB dissociates from NF-κB. Devoid of its natural inhibitor, the nuclear localization sequence of NF-κB allows translocation from the cytoplasm to the nucleus [78] (Figure 5).

In addition to the release from IκB and nuclear translocation, the activation of NF-κB requires a wide variety of post-translational modifications (PTMs) of NF-κB subunits including phosphorylation, acetylation, ubiquitylation, and methylation. These modifications control nuclear translocation, target gene specificity, transcriptional activity, and subunit degradation [79] and are essential for the transactivation of target genes beyond the simple expression levels or even subcellular localization. Phosphorylation of NF-κB, especially the transactivating REL associated (RelA)/p65 NF-κB isoform was investigated and multiple kinases were shown to be involved, conferring both activating and inhibiting phosphorylation [80,81,82,83,84] (Figure 5).

Moreover, oxidation or nitrosylation of both p50 and p65 subunits forming the NF-κB transcription factor are also known to inhibit DNA binding [85]. The conserved C-38 residue of p65 is the target of many naturally occurring NF-κB inhibitors such as the sesquiterpene lactones [86]. RelA C-38 is also subject to hydrogen sulfide-linked sulfhydration [87] performed by cystathionine-lyase (CSE). Many natural products covalently modify C-38 to modulate NF-κB activity.

Beyond methylation, RelA or p65 phosphorylation at S-276 facilitates the recruitment of p300/cAMP-response element-binding protein (CREB) binding protein (CBP). This phosphorylation of p65 leads to CBP/p300 general transcription machinery interaction required for transactivation [88].

**Figure 6:**
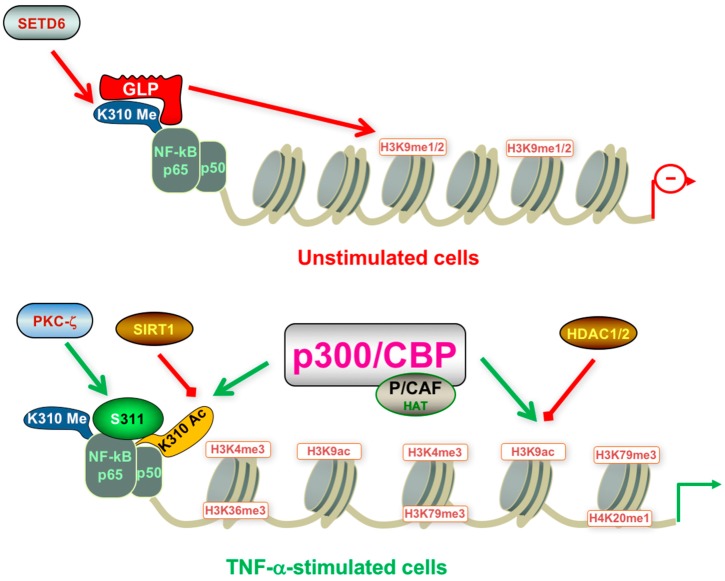
Regulation of posttranscriptional modifications of NF-κB and histone H3 with and without TNF-α stimulation. Open arrowheads designate addition of groups whereas closed arrowheads designate their removal; Red arrows show inhibiting post-translational modifications whereas green arrows show activating modifications, both related to NF-κB transactivation potential. cAMP-response element-binding protein binding protein (CBP); SET domain containing lysine methyltransferase 6 (SETD6); protein kinase C (PKC); G9a-like protein (methyltransferase) (GLP); p300/CBP-associated factor (P/CAF); histone acetyltransferases (HAT); histone deacetylase (HDAC); sirtuin (SIRT); tumor necrosis factor α (TNF-α); histone (H); methylation (me); acetylation (ac). (Scheme realized with Science Slides).

Post-translational modifications of NF-κB also include methylation of RelA/p65 leading to crosstalk between NF-κB and chromatin for the regulation of NF-κB target genes. In unstimulated cells, i.e., before TNF-α stimulation, the SET domain containing (SETD)6 methyltransferase methylates RelA at K-310 which triggers methylation of histone H3K9 by methyltransferase G9a-like protein (GLP) and leads to a transcriptionally repressed state of both chromatin and related NF-κB response genes. Furthermore, the crosstalk between methylated RelA and histone H3 maintains the repressed state of NF-κB target genes in unstimulated cells [89]. Whenever cells get stimulated by TNF-α, protein kinase (PK)C-ζ-mediates RelA phosphorylation at S-311 which abrogates methylation at K-310 and the binding of GLP. Recruitment of NF-κB co-activators and a change in the chromatin environment then eventually leads to transcription of NF-κB target genes. It is well known that the K-310me1 of RelA needs to be removed by a demethylase before p300/CBP accesses K-310 [89] (Figure 6).

More recently, a highly conserved cysteine residue was discovered in the N-terminal region of the Rel homology domain (RHD) (C-38 in human RelA—p65) [90]. This residue interacts with the phosphate backbone of NF-κB binding sites [91].

Recruitment of CBP eventually leads to acetylation of p65/RelA and nearby histones, leading to NF-κB gene expression [79]. Acetylation of p65 modulates DNA-binding capacity, transcriptional activity, interaction with IκB and subcellular localization [92,93]. Acetylation of RelA p65 at K-310 is critical for the transcriptional activation of NF-κB, but p65 is also acetylated by p300 at K-314 and K-315. Here, acetylation of K-314 is essential for late gene expression [94,95].

Site-specific acetylation of p65 by CBP controls the specificity of NF-κB-dependent gene expression. Reversible protein acetylation is an important post-translational modification that regulates the function of histones and other proteins including transcription factors [96]. Acetylation has a rapid turnover because of the highly dynamic equilibrium between HATs and HDACs.

Especially SIRT2 regulates NF-κB-dependent gene expression through deacetylation of p65 at K-310 [97]. SIRT2 has multiple functions and binds to HDAC6 to deacetylate α-tubulin contributing to microtubular dynamics and cell cycle regulation [98,99,100]. Moreover, SIRT2 deacetylates other proteins including p53 besides p300 and histones H3 and H4 substrates [101,102,103]. In fact, SIRT2 is localized mainly in the cytoplasm during G2–M transition: here this HDAC shuttles to the nucleus to deacetylate histone H4 at K-16 (H4K16) [101]. In SIRT2−/− cells p65 is hyperacetylated at K-310 after TNFα stimulation. Thus, expression of a subset of p65 acetylation-dependent target genes is increased. Altogether, p65 is deacetylated by SIRT2 in the cytoplasm to regulate the expression of specific NF-κB-dependent genes [97].

### 6.2. Synergistic Treatments Involving HDAC and NF-κB inhibitors

One hand increased acetylation levels of histones lead to upregulation of gene expression patterns and as such could also contribute not only to increased expression of cell cycle inhibitors including p21 but also to transactivation of NF-κB target genes. Such proteins are mostly involved in cell proliferation and resistance to cell death, two of the key hallmarks of cancer as defined by Hanahan and Weinberg [104].

Accordingly, a combination of both HDAC and NF-κB inhibitors could lead to synergistic effects in cancer types highly “addicted” to NF-κB and thus prevent unwanted pro-inflammatory side effects due to upregulation of pro-inflammatory or growth promoting gene products after HDACi treatment, considering the numerous NF-κB target genes. Such results were described after treatment of HeLa cells with the natural HDACis TSA (Figure 3) or apicidin (Table 3) triggering transcription of interleukin (IL)-8 and cellular inhibitor of apoptosis (cIAP)-1 thus rather contributing to the overall survival of cancer cells [105], abrogating the otherwise cell-death inducing capacity of apicidin. Here the authors demonstrated feasibility by using a genetic inhibitor (dominant negative IκB) to show that inhibition of NF-κB is strictly required to abrogate the pro-survival effect induced by apicidin (Table 3), eventually achieving sensitization of HeLa cells towards apoptosis. Similarly, up-regulation of NF-κB activity was shown in MCF-7 cells in the presence of TSA (Figure 3) [106].

**Figure 7:**
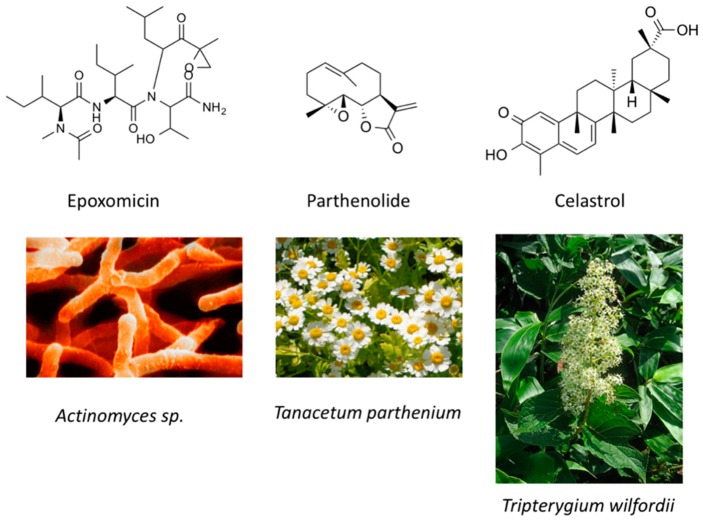
Molecular structures of natural compounds used in combination with histone deacetylases inhibitors.

In human colorectal cancer cells, a combination of apicidin (Table 3) and proteasome inhibitors MG132, proteasome inhibitor 1 or epoxomicin (Figure 7) inhibited cancer cell growth and induced apoptosis, decreased NF-κB activity and increased reactive oxygen species (ROS) production [107]. Similarly, TSA (Figure 3) and sodium butyrate (Figure 4) were shown to enhance activation of NF-κB and target genes by increasing NF-κB p50/p65 DNA binding and acetylation of the RelA p65. In this study, the authors used small interfering RNA against p65 or bortezomib, an inhibitor of proteasome-dependent NF-κB activation to increase cancer cell sensitization [108].

Similarly, SAHA (Figure 3) and bortezomib synergistically induce apoptosis in Mantle cell lymphoma cells [109] by a similar mechanism where the HDACi led to acetylated NF-κB whereas bortezomib abrogated this activation. In this study, apoptosis induction was accompanied by *N*-acetyl-l-cysteine-sensitized ROS production. Co-treatment of NF-κB inhibitor parthenolide (Figure 7) and vorinostat or LBH589 (Figure 3) similarly potentiated cell death in human AML cells through a process involving NF-κB inhibition [110].

In breast cancer, treatment of aromatase inhibitor-resistant cell lines with LBH589 (Figure 3) was also shown to suppress NF-κB1 mRNA (encoding the p105 subunit) and protein expression [111] even in the absence of additional NF-κB inhibitors. The combination of celastrol (Figure 7) and SAHA (Figure 3) exerted strong synergistic efficacy against human cancer cells in vitro and in vivo leading to caspase-mediated apoptosis [112].

Considering the multiple examples of synergistic effects of HDACi-mediated NF-κB activation coupled to a second compound known to block NF-κB cell signaling, Orlikova et al. suggested an approach with a single molecule with a dual inhibition potential. Accordingly, a series of hemi-synthetic chalcone lead compounds inhibited both TNF-α-induced NF-kB activity and total HDAC activity of classes I, II and IV (Table 3) providing insight into the future development of dual inhibitors in the treatment of inflammation and cancer [28].

Importantly, it was shown that HDAC modulators confer not only post-translational modifications to histones but also to many other cellular proteins, including NF-κB itself. NF-κB acetylation at K-310 is required for its transactivation potential. Accordingly, HAT lead to activation of NF-κB due to acetylation of this essential regulatory site whereas HDACs block NF-κB by its deacetylation. This dual regulatory potential leaves room for specific regulation steps that are intimately depending on the HDAC isoform activated or inhibited. For instance, SIRT1 was described to physically interact with RelA/p65 leading to transcriptional inhibition by deacetylating RelA/p65 at K-310 [113]. Interestingly natural compound resveratrol (Figure 3), a SIRT1 agonist, further activated NF-κB deacetylation, thus abrogating TNFα-induced transactivation of the NF-κB gene battery. Eventually, resveratrol leads to induction of apoptosis. Acetylated, activated, p65 is subsequently deacetylated via HDAC3, inducing binding to its inhibitor IκBα. Also in this instance pan-HDAC inhibition would prevent NF-κB deacetylation and likely contribute to the maintenance of its transactivation potential.

## 7. Conclusions and Perspectives

Results obtained so far clearly demonstrate the importance of specific HDACi to achieve a final cancer-inhibiting result, and that strict inhibition of HDAC is not the target in every instance. Therefore, HDAC isoform-specific inhibitors/activators will be of great importance combined with a better understanding of the essential role of both activating and inhibiting marks not only of histones but essentially also of other protein targets.

Accordingly, a combination treatment of selected highly specific HDAC inhibitors and activators would lead to an overall anticancer effect in a more rational way compared to the unspecific first generation pan inhibitors. Moreover, the cell type-specific effect of such inhibitors needs to be carefully considered as it was shown for compounds like apicidin that the effects can be pro-apoptotic in leukemia but apoptosis resistance-triggering in solid tumors.

Side effects observed with the first generation of pan-HDACi that are so far clinically approved could most likely be explained by the high simultaneous inhibition of HDACs with pro- and anticancer activities. Future research will aim at matching isoform-specific modulators with clearly identified cancer-specific molecular targets thus contributing to personalization of the cancer treatment. Considering the potent induction of NF-κB induction via hyperacetylation triggered by most pan-HDACi, pro-inflammatory effects are clearly foreseeable. Moreover, beyond NF-κB, Stat1 also requires acetylation on K-410 and K-413 [114] to interact further with NF-κB and certainly also contributes to pro-inflammatory signaling after HDACi treatment.

On the other hand, FR235222 selectively inhibits NF-κB activity rather than activating it so that many NF-κB-regulated genes were transcriptionally down-regulated in activated Jurkat T cells [115]. Considering the essential effect of HDACis on acetylation of target proteins, this inhibition is most likely indirect. The authors hypothesized that FR235222 abrogates interactions between NF-κB and coactivator proteins leading to NF-κB inhibition. Accordingly, future research needs to deeply investigate direct and indirect effects of novel and clinically used HDACi to prevent unwanted activation or inhibition mechanisms leading to adverse side effects. In that sense, the use of combined treatments against both HDACs and NF-κB seems interesting to trigger synergistic effects and abrogate side effects. Here the use of dual inhibitors appears to many as an excellent possibility to design entirely novel therapeutic approaches.

## Figures and Tables

**Table 1:** Histone deacetylase (HDAC) enzymes: classification, size, co-factor, cellular localization, physiological function, and expression.

Class	Members	Size (Amino Acids)	Co-Factor	Cellular Localization	Physiological Function	Expression
I	HDAC1	483	Zn^2+^	Nucleus	Cell proliferation and survival	Ubiquitous
	HDAC2	488	Zn^2+^	Nucleus	Cell proliferation and insulin resistance	
	HDAC3	428	Zn^2+^	Nucleus	Cell proliferation and survival	
	HDAC8	377	Zn^2+^	Nucleus	Cell proliferation	
IIa	HDAC4	1084	Zn^2+^	Nucleus/Cytoplasm	Control of cytoskeletal dynamics and cell mobility	Tissue-specific
	HDAC5	1122	Zn^2+^	Nucleus/Cytoplasm	Gluconeogenesis, cardiovascular growth, and function, and cardiac myocyte and endothelial cell function	
	HDAC7	912	Zn^2+^	Nucleus/Cytoplasm	Glycogenesis, endothelial function, and thymocyte differentiation	
	HDAC9	1069	Zn^2+^	Nucleus/Cytoplasm	Cardiovascular growth and function, homologous recombination, and thymocyte differentiation	
IIb	HDAC6	1215	Zn^2+^	Cytoplasm	Control of cytoskeletal dynamics and cell mobility	Tissue-specific
	HDAC10	669	Zn^2+^	Cytoplasm	Autophagy-mediated cell survival and homologous recombination	
III	SIRT1	747	NAD^+^	Nucleus/Cytoplasm	Autoimmune system regulation, cell survival, aging, and redox regulation	Variable
	SIRT2	389	NAD^+^	Nucleus	Cell survival, cell migration and invasion	
	SIRT3	399	NAD^+^	Mitochondria	Redox balance, ATP regulation and metabolism, urea cycle, apoptosis, and cell signaling	
	SIRT4	314	NAD^+^	Mitochondria	ATP regulation and metabolism, energy metabolism, apoptosis and cell signaling	
	SIRT5	310	NAD^+^	Mitochondria	ATP regulation, urea cycle, energy metabolism, apoptosis, and cell signaling	
	SIRT6	355	NAD^+^	Nucleus	Metabolic regulation	
	SIRT7	400	NAD^+^	Nucleus	Apoptosis	
IV	HDAC11	347	Zn^2+^	Nucleus	Immunomodulation and DNA replication	Ubiquitous

**Table 2:** Changes in histone deacetylase protein and gene expression implicated in cancer ^a^.

Class	Members	Type of Variation	Cancer Involved	References
I	HDAC1	Overexpression	Gastrointestinal carcinoma	[9]
			Prostate carcinoma	[9]
			Breast carcinoma	[9]
			Colon adenocarcinoma	[7]
			Chronic lymphocytic leukemia	[10]
		*HDAC1* overexpression	Ovarian cancer	[4]
			Gastric cancer	[4]
			Hodgkin’s lymphoma	[4]
			Prostate cancer	[4]
		*HDAC1* downregulation	Colorectal cancer	[4]
	HDAC2	Overexpression	Uterine cancer	[9]
			Cervical cancer	[9]
			Gastric cancer	[9]
		*HDAC2* overexpression	Ovarian cancer	[4]
			Hodgkin’s lymphoma	[4]
		Truncating *HDAC2* mutation	Colon cancer	[4]
			Endometrial cancer	[4]
			Gastric cancer	[4]
	HDAC3	Overexpression	Colon cancer	[9]
			Hodgkin’s lymphoma	[4]
			Chronic lymphocytic leukemia	[10]
		*HDAC3* overexpression	Ovarian cancer	[4]
			Lung cancer	[4]
IIa	HDAC4	*HDAC4* mutations	Breast cancer	[4]
			Colorectal cancer	[4]
		*HDAC4* overexpression	Prostate cancer	[4]
			Breast cancer	[4]
		*HDAC4* downregulation	Colon cancer	[16]
			Lung cancer	[16]
	HDAC5	*HDAC5* downregulation	Colorectal cancer	[4]
	HDAC7	Overexpression	Chronic lymphocytic leukemia	[10]
		*HDAC7* overexpression	Colorectal cancer	[4]
			Pancreatic cancer	[4]
	HDAC9	Overexpression	Chronic lymphocytic leukemia	[10]
IIb	HDAC6	Overexpression	Acute lymphoblastic leukemia	[11]
			Acute myeloid leukemia	[11]
			Breast cancer	[11]
			Chronic lymphocytic leukemia	[11]
			Cutaneous T-cell lymphoma	[11]
			Hepatocellular carcinoma	[11]
			Oral squamous cell carcinoma	[11]
			Ovarian cancer	[11]
			Urothelial cancer	[11]
	HDAC10	Overexpression	Chronic lymphocytic leukemia	[10]
III	SIRT1	Overexpression	Acute myeloid leukemia	[12]
			Skin cancer	[12]
			Colon cancer	[12]
			Prostate cancer	[12]
			Chronic lymphocytic leukemia	[10]
		*SIRT1* downregulation	Colorectal cancer	[13]
	SIRT6	Overexpression	Chronic lymphocytic leukemia	[10]
		Downregulation	Liver cancer	[14]
	SIRT7	Overexpression	Breast cancer	[15]

^a^ In this table, HDAC in italics designate the corresponding gene.

**Table 3:** Natural compounds with HDAC inhibitory function.

Compound Name	Chemical Category	HDAC Specificity	Origin of the Compound	Structure	Organism	Ref.
Lycorine	Alkaloid	Pan HDAC	*Amaryllidaceae*	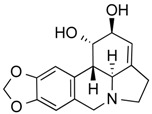	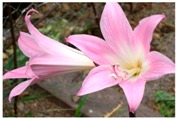	[42]
Burkholdac A	Depsipeptide	Class I HDAC	*Burkholderia thailandensis*	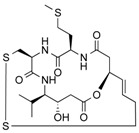	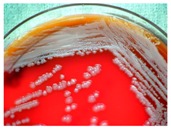	[43]
Largazole	Depsipeptide	Class I HDAC HDAC1, 2, 3	*Cyanobacterium Symploca* sp.	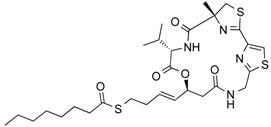	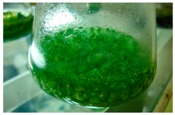	[44]
Spiruchostatin A (YM753)	Depsipeptide	Class I HDAC	*Pseudomonas* sp.	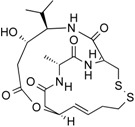	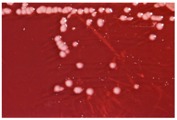	[45]
Thailandepsin A (Burkholdac B)	Depsipeptide	Class I HDAC	*Burkholderia thailandensis*	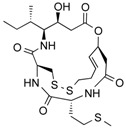	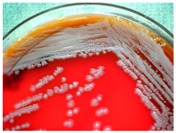	[46,47]
9-Hydroxystearic acid	Fatty acid	Class I HDACs	Endogenous lipid peroxidation by-product	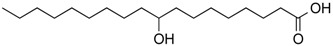	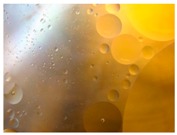	[48]
Amamistatin B	Mycobacterial siderophore	Pan HDAC	*Nocardia* sp.	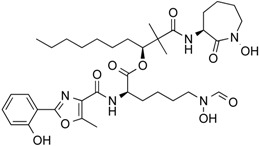	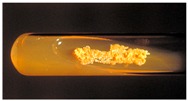	[49]
Diallyl disulfide	Organosulfur compound	Increased acetylation	*Allium sativum*	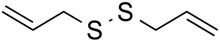	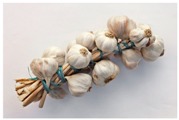	[50]
(*S*)-allylmercaptocysteine	Organosulfur compound	Increased acetylation	*Allium sativum*	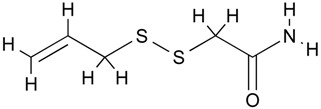	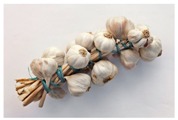	[51]
Sulforaphane	Organosulfur compound	Increased acetylation	*Brassica oleracea*	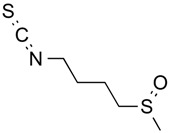	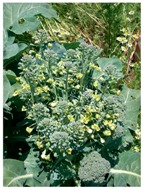	[52]
Aceroside VIII	Phenolic	HDAC6	*Betula platyphylla*	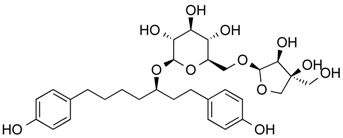	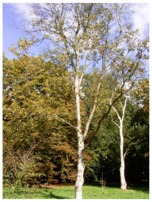	[53]
Butein	Phenolic	HDAC classes I, II and IV	*Toxicodendron vernicifluum*	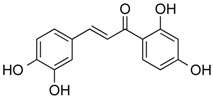	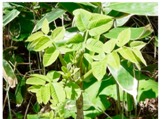	[28]
Homobutein	Phenolic	HDAC classes I, II and IV	*Butea frondosa*	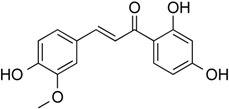	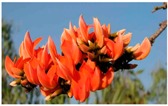	[28]
Isoliquiritigenin	Phenolic	HDAC classes I, II and IV	*Glycyrrhiza glabra*	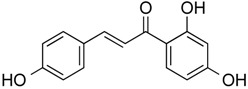	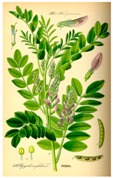	[28]
Kaempferol	Phenolic	HDACs of class I, II and IV	*Aloe vera*	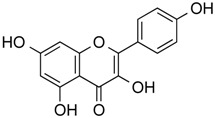	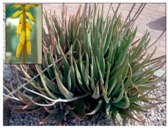	[54]
Marein	Phenolic	HDAC classes I, II and IV	*Coreopsis maritima*	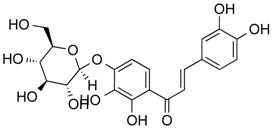	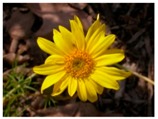	[28]
Protocatechuic aldehyde	Phenolic	HDAC2	*Hordeum vulgare*	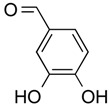	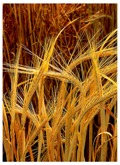	[55]
Psammaplin A	Phenolic	Class I HDAC	*Poecillastra* sp. and *Jaspis* sp.	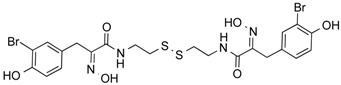	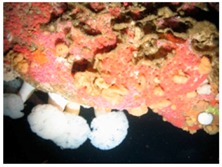	[56]
Resveratrol	Phenolic	HDACs of class I, II and IV	*Vitis vinifera*	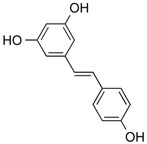	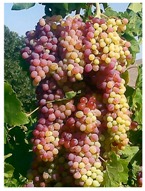	[57]
Sinapinic acid	Phenolic	Pan HDAC	*Hydnophytum formicarum* Jack.	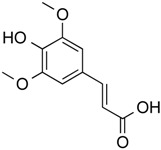	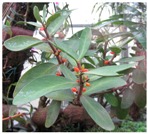	[58]
Depudecin	Polyketide	HDAC1	*Alternaria brassicicola*	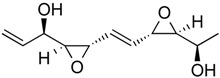	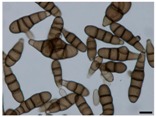	[59]
Epicocconigrone A	Polyketide	HDACs of class I, IIb and IV	*Epicoccum nigrum*	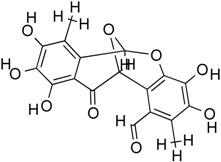	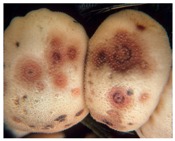	[60]
20(*S*)-Ginsenoside Rh_2_	*Steroid glycoside*	HDAC1, HDAC2, HDAC6	*Panax ginseng*	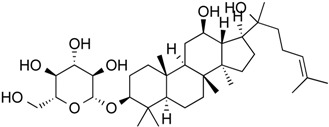	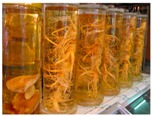	[61]
6-methoxy-2*E*,9*E*-humuladien-8-one	Terpenoid (sesqui-)	Pan HDAC	*Zingiber zerumbet* (L.) J. E. Smith	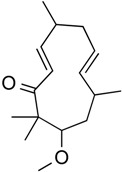	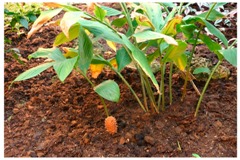	[62]
Zerumbone	Terpenoid (sesqui-)	Pan HDAC	*Zingiber zerumbet* (L.) J. E. Smith	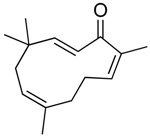	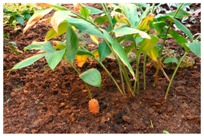	[62]
β-Thujaplicin (hinokitiol)	Terpenoid (mono)	HDAC2	*Cupressaceae* sp.	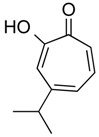	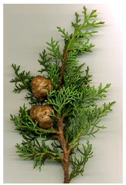	[63]
β-boswellic acid derivatives	Terpenoid (pentacyclic tri-)	HDAC8	*Boswellia sacra*	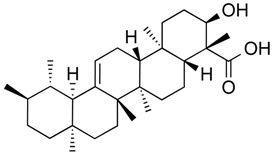	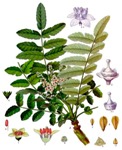	[64]
Apicidin	Tetrapeptide	Class I HDAC HDAC1, 2, 3, 8	*Fusarium* sp.	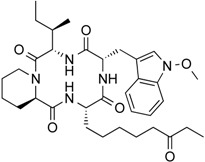	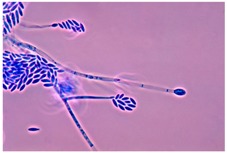	[65]
Azumamide E	Tetrapeptide	Class I HDAC HDAC1, 2, 3	*Mycale izuensis*	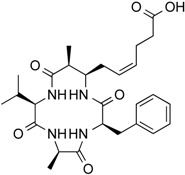	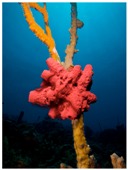	[66]
Chlamydocin	Tetrapeptide	HDAC1, 6	*Diheterospora chlamydosporia*	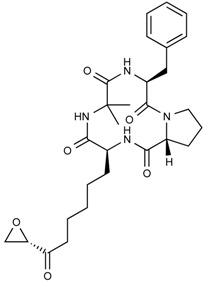	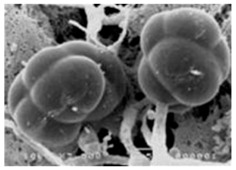	[67]
Trapoxin A	Tetrapeptide	Class I HDAC	*Helicoma ambiens* RF-1023	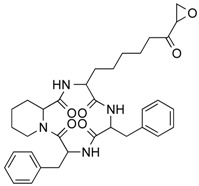	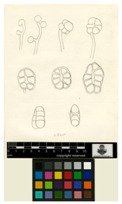	[68,69]
Trapoxin B	Tetrapeptide	Class I HDAC	*Helicoma ambiens* RF-1023	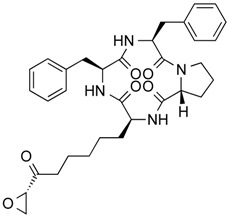	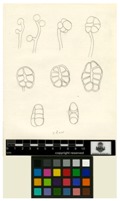	[68,69]

## Abbreviations

The following abbreviations are used in this manuscript:

ALL	acute lymphoblastic leukemia
AML	acute myeloid leukemia
CBP	CREB binding protein
cIAP	cellular inhibitor of apoptosis
CLL	chronic lymphocytic leukemia
CREB	cAMP-response element-binding protein
CSE	cystathionine-lyase
CTCL	cutaneous T-cell lymphoma
GLP	G9a-like protein
HAT	histone acetyltransferase
HAT	histone acetyltransferases
Hda1	histone deacetylase 1
HDAC	histone deacetylase
HDACi	histone deacetylase inhibitor
HMBA	hexamethylene bisacetamide
IKK	IκB kinase
IL	interleukin
INF	interferon
IκB	inhibitor of κB
MDS	myelodysplastic syndromes
MDS	myelodysplastic syndromes
MEKK	mitogen-activated protein kinase kinase kinase
NAD	nicotinamide adenine dinucleotide
NEMO	NF-κB essential modulator
NF-κB	nuclear factor-κB
P/CAF	P300/CBP-associated factor
PK	protein kinase
PTCL	peripheral T-cell lymphoma
RelA	REL associated
RHD	Rel homology domain
RIP	receptor-interacting protein
ROS	reactive oxygen species
Rpd3	reduced potassium dependency-3
SAHA	suberoylanilide hydroxamic acid
SCF	Skp, Cullin, F-box (containing complex)
SETD	SET domain containing
Sir2	silent information regulator-2
SIRT	sirtuin
TAB	TGF-β-activated kinase 1 and MAP3K7-binding protein
TAK	transforming growth factor β-activated kinase
TNF	tumor necrosis factor
TGF	tumor growth factor
TRADD	tumor necrosis factor receptor type 1-associated death domain
TRAFF	TNF receptor-associated factor
TSA	trichostatin A
Ubc	Ubiquitin-conjugating (enzyme)
USFDA	United States Food and Drug Administration
VPA	valproic acid
ZBD	Zn^2+^ binding domain
